# A hybrid approach for binary and multi-class classification of voice disorders using a pre-trained model and ensemble classifiers

**DOI:** 10.1186/s12911-025-02978-w

**Published:** 2025-05-01

**Authors:** Mehtab Ur Rahman, Cem Direkoglu

**Affiliations:** 1 Department of Language and Communication, Radboud University, Houtlaan, Nijmegen, Gelderland 6525 Netherlands; 2 Electrical and Electronics Engineering Department, Middle East Technical University, Northern Cyprus Campus, Kalkanli, Güzelyurt, Mersin 10 99738 Turkey

**Keywords:** Voice disorders, Multi-class classification, Ensemble classifier, VGGish

## Abstract

Recent advances in artificial intelligence-based audio and speech processing have increasingly focused on the binary and multi-class classification of voice disorders. Despite progress, achieving high accuracy in multi-class classification remains challenging. This paper proposes a novel hybrid approach using a two-stage framework to enhance voice disorders classification performance, and achieve state-of-the-art accuracies in multi-class classification. Our hybrid approach, combines deep learning features with various powerful classifiers. In the first stage, high-level feature embeddings are extracted from voice data spectrograms using a pre-trained VGGish model. In the second stage, these embeddings are used as input to four different classifiers: Support Vector Machine (SVM), Logistic Regression (LR), Multi-Layer Perceptron (MLP), and an Ensemble Classifier (EC). Experiments are conducted on a subset of the Saarbruecken Voice Database (SVD) for male, female, and combined speakers. For binary classification, VGGish-SVM achieved the highest accuracy for male speakers (82.45% for healthy vs. disordered; 75.45% for hyperfunctional dysphonia vs. vocal fold paresis), while VGGish-EC performed best for female speakers (71.54% for healthy vs. disordered; 68.42% for hyperfunctional dysphonia vs. vocal fold paresis). In multi-class classification, VGGish-SVM outperformed other models, achieving mean accuracies of 77.81% for male speakers, 63.11% for female speakers, and 70.53% for combined genders. We conducted a comparative analysis against related works, including the Mel frequency cepstral coefficient (MFCC), MFCC-glottal features, and features extracted using the wav2vec and HuBERT models with SVM classifier. Results demonstrate that our hybrid approach consistently outperforms these models, especially in multi-class classification tasks. The results show the feasibility of a hybrid framework for voice disorder classification, offering a foundation for refining automated tools that could support clinical assessments with further validation.

## Introduction

Voice production is the process by which humans produce sound to communicate ideas, meaning, opinions, and other information. The human voice production system includes the lungs, larynx, vocal tract, and vocal folds. The lungs provide the air pressure that is needed to vibrate the vocal folds. The vocal folds are located in the larynx, also known as the voice box. When the vocal folds vibrate, they create sound waves that travel through the vocal tract, which is the passage of air from the larynx to the mouth and nose. The shape of the vocal tract affects the timbre of the voice. Voice disorders can occur when there is a problem with any of the components of the voice production system causing changes in the pitch, loudness, or quality of the voice. These disorders can reduce the clarity of a person’s oral communication ability. Voice disorders can vary in severity from minor hoarseness or alterations in vocal quality to the extreme outcome of complete voice loss [1].

Voice disorders can result from various factors. These disorders are commonly classified based on their underlying causes, which may include psychogenic, functional, or organic factors. Organic voice disorders are caused by structural or neurological problems that affect the vocal folds or other parts of the voice production system [2]. Functional voice disorders occur when the vocal mechanism is not used efficiently, even though the physical structure of the larynx and vocal tract is normal. Psychogenic voice disorders, on the other hand, stem from psychological factors such as sadness, anxiety, or emotional responses to traumatic or stressful situations [3].

Voice disorders can have a significant impact on people of all ages, potentially leading to stress, embarrassment, frustration, withdrawal, and depression. Professions that require frequent and demanding use of the voice, such as teaching, acting, and singing, are particularly susceptible to these disorders [4]. To ensure the right treatment, accurate classification of the voice disorders is crucial. A speech therapist typically evaluates the patient’s voice quality for this purpose. However, this approach is subjective and relies on the speech therapist’s expertise. Another approach to assess voice disorders is to use artificial intelligence (AI) to process acoustic features of voice signals, which provides an objective assessment. Automatic classification of voice disorders can provide speech therapists with a faster and more comfortable way to identify voice disorders in patients.

Recent advances in AI have enabled significant progress in audio and speech processing tasks, including speaker identification, speech emotion recognition, and voice disorder detection. For instance, Xie et al. [5] employed attention-based long short-term memory (LSTM) networks to classify speech emotions. Similarly, Keser and Gezer [6] conducted a comparative analysis of speaker identification methods, combining deep learning, machine learning, and subspace classifiers with diverse feature extraction techniques. Authors in [7] further demonstrate the potential of deep multiple instance learning for voice activity detection (VAD). These studies highlight the versatility of hybrid approaches—integrating feature engineering with classifiers like support vector machines (SVMs), logistic regression, and neural networks—to address the unique challenges of audio classification. However, voice disorder classification poses distinct difficulties due to the subtle acoustic variations between disorders and the need for high diagnostic precision. While existing works often focus on binary classification, multi-class frameworks remain underexplored. This gap motivates our proposed framework, which combines deep learning-based feature extraction with robust classifiers to improve both binary and multi-class classification performance.

### Related work

In recent years, researchers have become increasingly interested in the automatic classification of voice disorders. These methods, utilizing computer algorithms to analyze speech signals, can revolutionize the classification and detection of voice disorders, making it more objective, efficient, and accessible. Most researchers focus on binary classification problems, such as classifying between healthy and pathological voices or the detection of a voice disorder. Spectrograms and cepstral analysis are two commonly used features for this purpose. However, in recent years, machine learning algorithms have gained popularity for their ability to learn and recognize patterns in acoustic features associated with various types of voice disorders. Fang et al. [8] used Mel Frequency Cepstral Coefficients (MFCCs) and three classifiers, namely Deep Neural Network (DNN), Gaussian Mixture Model (GMM) and SVM, for pathological voice detection. Cordeiro et al. [9] applied hierarchical classification for the identification of pathological voice, employing MFCCs and line spectral frequencies features. Kodrasi et al. [10] proposed a hierarchical multi-class automatic technique using handcrafted acoustic features to distinguish between speech apraxia, dysarthria and neurotypical speech. The approach utilizes two SVMs, with the first SVM distinguishing between neurotypical speech and impaired, while the second SVM discriminates between dysarthria and apraxia of speech.

Costa et al. [11] proposed combining the hidden Markov model (HMM) and modified MFCCs for the voice disorders caused by a vocal fold pathology. In [12, 13], the authors applied multilayer neural networks for the classification of MFCC features and demonstrated that results can be enhanced by considering the differentiation of the speaker’s gender. Ali et al. [14] introduced a method for the classification and detection of voice disorders, utilizing a Gaussian mixture model (GMM) classifier with running speech voice data. Benba et al. [15] investigated the detection of dysphonia using a Naive Bayes (NB) algorithm. They extracted acoustic features using MFCC. Authors in [16] used MFCC features to differentiate between Parkinson’s disease (PD) and healthy voices. They extracted MFCC features from three different vowel sounds: /a/, /o/, and /u/. Authors in [17, 18] also explored binary classification of voice disorders in their research. In [19], the authors employ wavelet scattering features to capture both time-frequency information from voice signals, which are then used for classifying neurological voice disorders.

In addition to binary classification, the majority of research studies use sustained vowel /a/ recordings from clinical settings for their investigations [20]. In [21], the introduction of continuous speech and vowel /a/ analysis for voice disorder identification is discussed. The authors conducted a comparison of glottal features extracted from the sustained vowel sound /a/ and voiced segments within continuous speech. Fujimura et al. [22] used an end-to-end 1D-CNN model to classify voice disorders using voice samples of the sustained vowel /a/. The research demonstrated that the 1D-CNN models were capable of consistently evaluating voice disorders, aligning with human assessments.

In recent years, deep learning has achieved impressive results in a variety of areas, including natural language processing, computer vision and audio analysis. Deep learning’s ability to handle complex and high-dimensional acoustic features makes it well-suited for addressing the challenges of voice disorders classification. This has encouraged many researchers to explore the potential of deep learning for voice disorder classification. Wu et al. [23] developed a novel system using spectrograms of disordered and normal speech recordings as input. They employed Convolutional Deep Belief Networks for pre-training CNN weights as a generative model to understand the input data’s structure statistically. Subsequently, they fine-tuned the CNN using supervised back-propagation. In [24], authors propose the use of a CNN model along with short-time Fourier transform (STFT) features for the binary classification of voice disorders. Mohammed et al. [25] addressed the problem of voice disorder detection by using CNN model. They specifically focused on the automatic detection of depression from speech. Chaiani et al. [26], the authors analyzed an algorithm that extracts a chromagram acoustic feature from voice samples and uses it as input to a CNN-based classification system. The research in [27] proposed a two-stage framework for the classification of different voice disorders. The first stage uses speech enhancement to improve the voice signal quality by removing noise. The second stage employs a CNN with long short-term memory (CNN-LSTM) to learn complex features from spectrograms of the enhanced voice signals. Harar et al. [28] proposed a novel approach for voice pathology detection that uses convolutional and LSTM layers to learn directly from raw audio signals. Furthermore, recent studies have highlighted the beneficial impact of denoising for audio signals [29], advanced vocal feature extraction [30]. These approaches collectively suggest promising avenues for enhancing the automatic classification of voice disorders.

In voice disorders classification, limited data availability is a common challenge. To address this, some researchers have used pre-trained models [31–34]. In [35], the authors proposed a transfer learning framework that uses a pre-trained OpenL3-SVM model and linear local tangent space alignment (LLTSA) for dimensionality reduction. They first extracted the Mel spectrum of the voice signals and then fed it into the OpenL3 model to obtain high-level feature embeddings. Violeta et al. [36] investigated the performance of self-supervised pre-trained Wav2Vec 2.0 and WavLM models for automatic pathological speech recognition using different setups. Zhu et al. [37, 38] introduced pre-trained BERT and WavBERT models for the detection of dementia using human speech. Karaman et al. [39] employed the SqueezeNet1_1, ResNet101, and DenseNet161 networks for the detection of Parkinson’s disease based on speech signals. The findings showed that the proposed networks, which utilize pre-trained models with a fine-tuning approach, achieved promising results. In [40], the authors used a pre-trained ResNet50 model for dysarthric speech detection.

### Research gap and contribution

Most studies on the automatic classification of voice disorders have focused on the binary classification, typically distinguishing between pathological and healthy voices. Some studies have taken a more specialized approach, aiming to identify particular pathological voices among all other pathological and healthy voices. A few studies have investigated multi-class classification of voice disorders, but the accuracy of these approaches is low. Multi-class classification of voice disorders is a challenging problem due to the limited training data and subtle differences between different types of disordered voices. In this study, we address both binary and multi-class classification of voice disorders. For binary classification, we distinguish between healthy and disordered voices, as well as between two different types of pathological voices. For multi-class classification, we have three classes: healthy, vocal fold paresis and hyperfunctional dysphonia.

Gender-specific classification of voice disorders has not been widely investigated. We present classification results separately for male and female speakers, as well as combined results, for both binary and multi-class tasks. This enables us to analyze and compare gender-based differences in the classification of voice disorders.

Feature extraction is a crucial step in machine learning tasks, and it holds particular significance in the classification of voice disorders due to the small dataset size. We utilize the pre-trained VGGish model [41] to extract 128-dimensional high-level embedding features using logarithmic mel spectrogram of voice data. As the name indicates, the VGGish network takes inspiration from a well-known VGG network and is adapted for audio classification. This model was trained on a large Audio set, which was a preliminary version of the YouTube-8M dataset. These embeddings are then utilized as input for machine learning classifiers.

Previous studies have employed transformer-based models like wav2vec and HuBERT for extracting audio embeddings. While these models perform well in general speech tasks, we found that VGGish, a CNN-based model, delivers better results for the classification of voice disorders. Consequently, our approach outperforms transformer-based models in this domain.

Our dataset is imbalanced, which mirrors the distribution often seen in real-world applications, where certain voice disorders are less common. This imbalance presents challenges in accurately classifying minority classes. To overcome this issue, we employed ensemble classifiers that combine the strengths of multiple models, improving performance on minority classes and enhancing overall classification accuracy.

We tested three machine learning classifiers: Logistic Regression (LR), Multi-Layer Perceptron (MLP), and Support Vector Machine (SVM). We also employed an ensemble classifier (EC) using SVM, LR, and MLP with soft voting to combine the predictions of the three classifiers. This allowed us to leverage the collective insights of these diverse classifiers and improve the overall classification performance.

This study demonstrates the effectiveness of utilizing embeddings from a pre-trained VGGish model and ensemble classifiers for both binary and multi-class classification of voice disorders. Additionally, we examine the impact of gender on the classification task. Our findings are compared to popular baseline methods, providing a comprehensive evaluation of our approach. The results show that our method outperforms the baseline approaches on both binary and multi-class classification tasks, demonstrating the superiority of the proposed method.

### Paper outline

The rest of the paper is structured as follows. Section “Proposed method” presents an in-depth explanation of the proposed method. Section “Dataset and experimental setup” describes the experimental setup and the voice dataset used in this study. Section “Experiments and results” provides a comprehensive overview of the experiments conducted for both binary and multi-class classification tasks. We present the results and performance metrics achieved by our approach. Section “Discussion” presents the implications of our findings. Section “Conclusion” summarizes the key points and highlights the main contributions made by our study.

## Proposed method

**Fig. 1:**
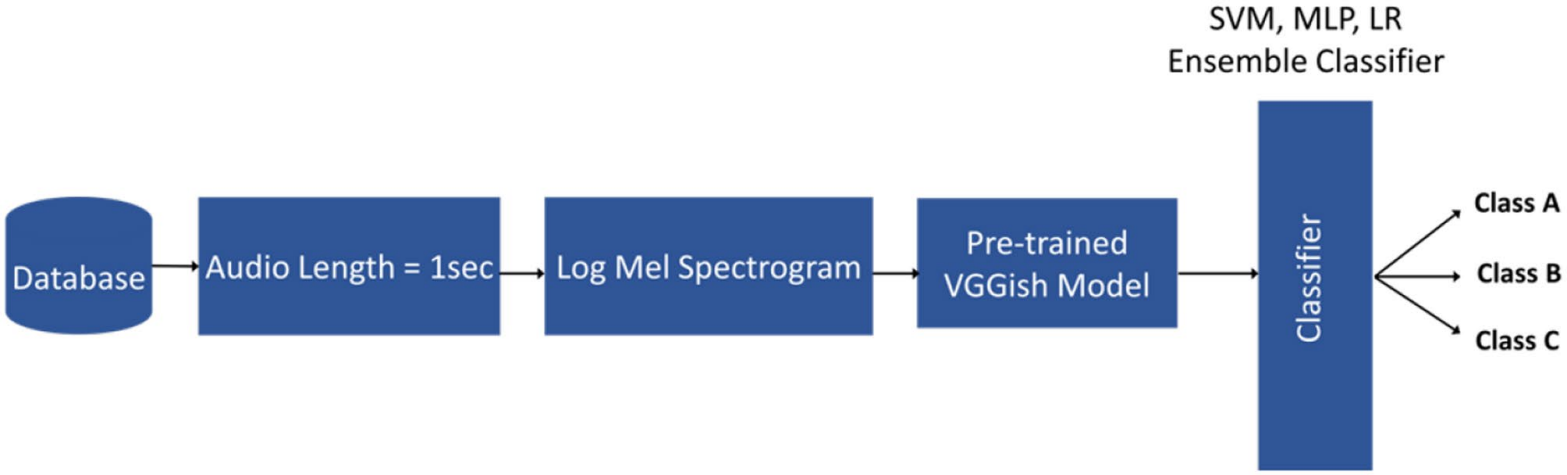
The proposed voice disorders classification system

In this paper, we propose a novel hybrid two-stage framework for voice disorders classification. In the first stage, voice data is converted into logarithmic mel spectrograms and high-level feature embeddings are extracted from these spectrograms using the pre-trained VGGish model. In the second stage, we use classifiers, including an ensemble classifier, to classify the feature embeddings. Figure 1 provides an illustration of the proposed classification framework.

### Preprocessing and feature extraction

Before extracting features, we apply several preprocessing steps. In the original dataset, as explained in Sect. 3, voice signals were recorded at 50 kHz sampling frequency. To align with our processing requirements, we resampled the audio to 16 kHz. The length of each audio recording in the original database is different. We trimmed the audio signals to 1 s. Audio signals that were less than 1 s were padded with zeros to ensure that all audio data had the same length.

The VGGish model takes the logarithmic mel spectrogram of an audio signal for feature extraction. To compute the mel spectrogram for each audio, we apply the Short-Time Fourier Transform (STFT) with a Hamming window lasting 25 milliseconds (ms) and a 10 ms shift. This resultant spectrogram is subsequently integrated into 64 frequency bins spaced along the Mel scale, and the magnitude of each bin is then transformed logarithmically. The configuration of the mel spectrogram draws inspiration from psychoacoustic analysis, which strives to replicate characteristics of the human auditory system. This procedure involves the application of a Mel filter bank denoted as *H*_m_(*k*) to filter the spectral line energy of the audio. The purpose of these filters is outlined by the following equations.

1 $${H_m}(k) = \left\{ {\matrix{ 0 & {{\text{if }}k < f(m - 1)} \cr {{{k - f(m - 1)} \over {f(m) - f(m - 1)}}} & {{\text{if }}f(m - 1) \le k \le f(m)} \cr {{{f(m + 1) - k} \over {f(m + 1) - f(m)}}} & {{\text{if }}f(m) < k \le f(m + 1)} \cr 0 & {{\text{if }}k > f(m + 1)} \cr } } \right.$$

Here, 0 ≤ *m* ≤ *M*, and *M* represents the count of filters. The central frequency *f*(*m*) of the filters can be written as:

2 $$f(m) = \left( {\frac{N}{{fs}}} \right)F_{mel}^{ - 1}\left( {{F_{mel}}({f_l}) + m\frac{{{F_{mel}}({f_h}) - {F_{mel}}({f_l})}}{{M + 1}}} \right)$$

Here, *f*_*l*_ denotes the lowest frequency within the filter’s frequency domain. *f*_*h*_ represents the highest frequency. *N* corresponds to the length of the Fourier transform. *f*_*s*_ stands for the sampling frequency. *F*_*mel*_ signifies the Mel frequency. The transformation formula linking *F*_*mel*_ and the regular frequency *f* is given by:

3 $${F_{mel}} = 2595\log \left( {1 + \frac{f}{{700}}} \right)$$

The log mel spectrogram tensor (96 × 64) is the input to VGGish. Here, 96 is the number of frames within each time scale, and 64 is the number of frequency bands.

**Fig. 2:**
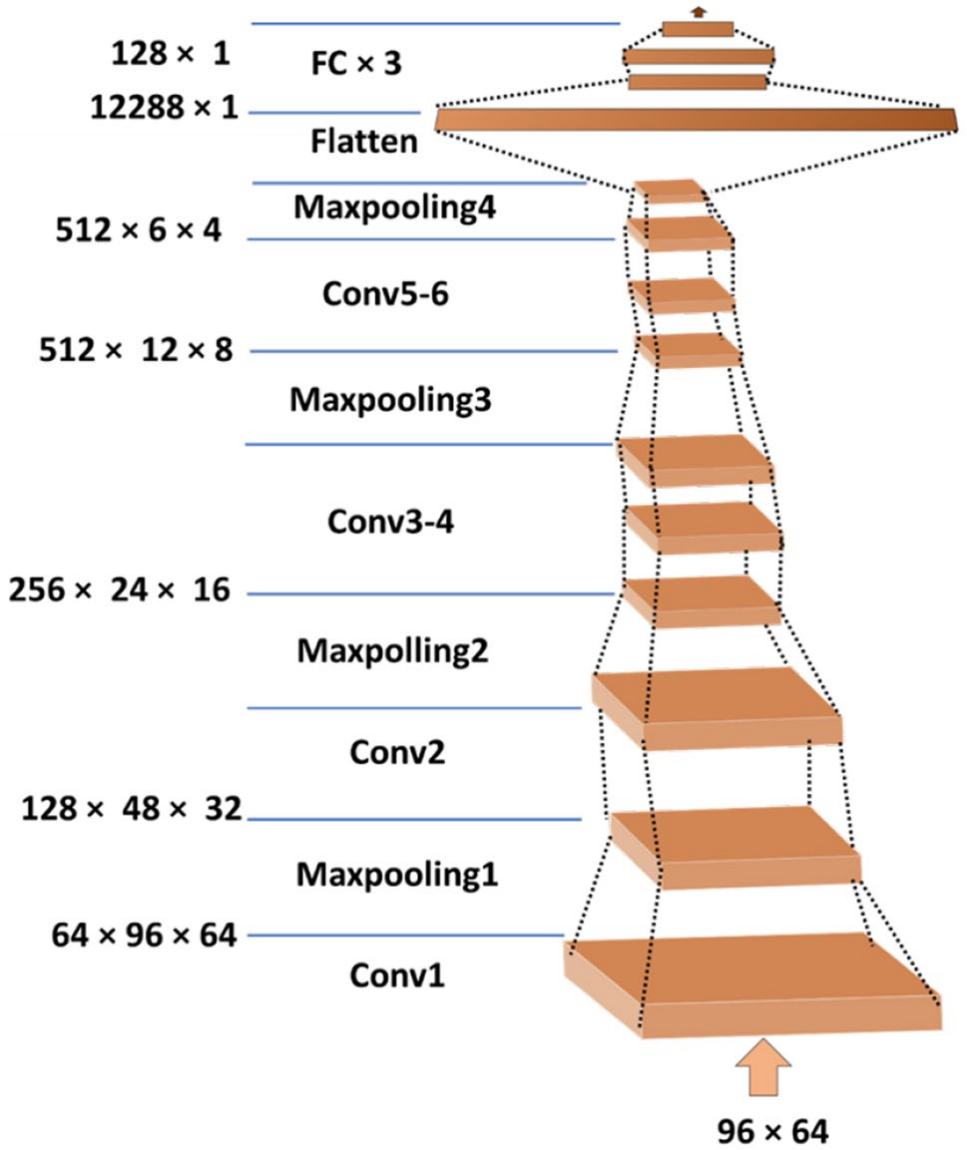
VGGish model architecture

In Fig. 2, the VGGish [41] model’s structure is illustrated. Batch normalization was implemented following each convolutional layer. The chosen loss function was cross-entropy, and the model employed the Adam optimizer. Dropout, weight decay, and other usual regularization methods were not utilized. This architecture was trained on a large Audio set which was a preliminary version of the YouTube-8 M dataset. We extracted 128-dimensional high-level feature embeddings using a pre-trained VGGish model. These embeddings are then utilized as input for machine learning classifiers.

### Classifiers

We evaluated the performance of three classifiers: SVM, LR, and MLP. The SVM classifies audio signals by mapping high-dimensional VGGish features into a new space using a kernel, allowing it to create a nonlinear decision boundary. LR assigns weight coefficients to features and makes predictions based on probability scores. The MLP model learns hierarchical representations through its hidden layers, capturing complex patterns in the VGGish embeddings.

**Fig. 3:**
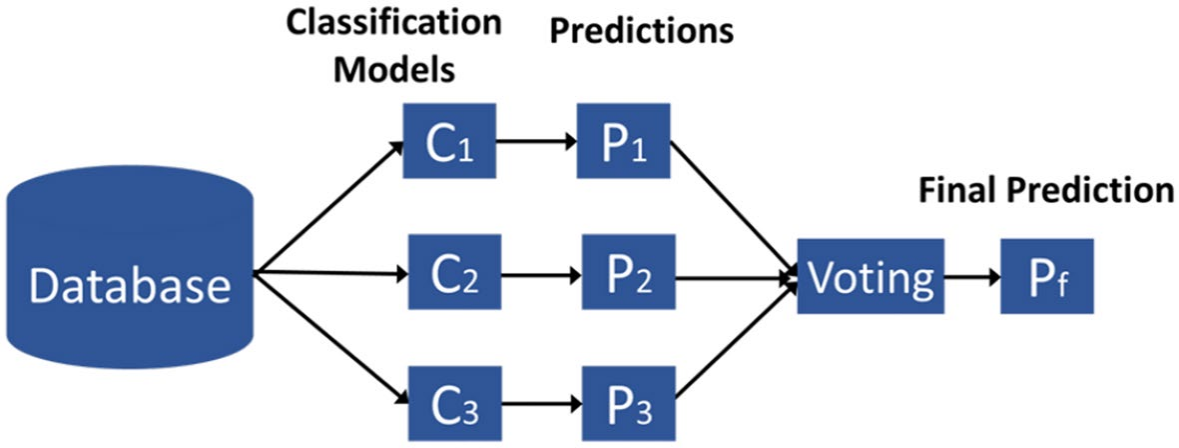
Ensemble classifier

In addition, we utilized an ensemble classifier (EC) that incorporated SVM, LR, and MLP. Figure 3 shows the EC model. Instead of relying on a single model, the EC combines the predictions of multiple models to improve accuracy and reduce the risk of overfitting. Soft voting was employed to combine the predictions of these three classifiers. Soft voting is an ensemble strategy that combines the predictions of multiple classifiers by averaging their predicted probability scores for each class. In soft voting, each classifier outputs a probability distribution over the classes. For a given input sample, let *p*_*i*_(*c*) be the probability that classifier *i* assigns to class *c*. With *K* classifiers, the ensemble probability for class *c* is computed as:

4 $$P(c|x) = \frac{1}{K}\sum\limits_{i = 1}^K {p_i}(c)$$

Then, the final predicted class is the one with the highest averaged probability.

5 $$\hat y = \arg \mathop {\max }\limits_c P(c|x) = \arg \mathop {\max }\limits_c \left( {\frac{1}{K}\sum\limits_{i = 1}^K {p_i}(c)} \right)$$

While the EC demonstrated superior performance for female speakers in the binary classification task (see Sect. 4), SVM emerged as the top-performing individual model for multiclass classification across genders and for male speakers in binary classification. This advantage can be attributed to SVM’s ability to capture complex, nonlinear relationships within high-dimensional VGGish features while maintaining robust generalization through rigorous regularization.

In our experiments, we employed SVM with a radial basis function (RBF) kernel. The SVM was configured with a regularization parameter of ‘1’, ‘scale’ kernel coefficient and utilized the ‘ovr’ (one-vs-rest) decision function shape. A logistic regression classifier with a maximum iteration count of 300, ‘newton-cg’ solver, L2 penalty and ‘ovr’ multi-class strategy was utilized to ensure convergence and prevent overfitting of the training data. Furthermore, we incorporated an MLP classifier with two hidden layers, stochastic gradient descent solver, a learning rate of 0.001 and ReLU activation function. To optimize the classifier’s performance, we employed the grid search technique. We tested all classifiers for male and female speakers separately, as well as combined for both binary and multi-class tasks.

## Dataset and experimental setup

This section provides a comprehensive description of the voice dataset used in the study. We also describe the training and testing process of classifiers.

### Dataset

We selected a subset of voice data from the publicly available Saarbruecken Voice Database (SVD) [42, 43] for this study. The SVD database was created by researchers at the Institut für Phonetik at Saarland University and the Phoniatry Section of the Caritas Clinic St. Theresia in Saarbrücken. The database contains audio recordings of 71 different voice disorders. Speakers engage in various speaking tasks, including the pronunciation of vowels ‘a’, ‘i’, and ‘u’ at normal, high, low, and rising-falling pitches as well as saying the sentence “Guten Morgen, wie geht es Ihnen?” (“Good morning, how are you?”). This includes individuals who were recorded before and after recovery from a voice disorder. Every recording in the database was captured at a sampling frequency of 50 kHz and 16 bits resolution.

We extracted a subset from the SVD database containing three classes: healthy, hyperfunctional dysphonia, and vocal fold paresis. The healthy class includes 227 recordings of males and 360 recordings of females. The hyperfunctional dysphonia voice disorder class has 32 recordings of males and 114 recordings of females. The vocal fold paresis voice disorder class has 25 recordings of males and 60 recordings of females. We included the recordings from individuals whose ages ranged from 19 to 60 years at the time of recording. Table 1 provides details of the subset used in this work. We chose hyperfunctional dysphonia and vocal fold paresis because they are commonly found voice disorders [44]. By choosing these specific disorders and matching the number of recordings used in previous study [45], we were able to directly compare our experimental results with existing research. This approach allowed for a more robust and fair evaluation of our findings.

**Table 1:** Details of voice recordings for each class

Class	Male recordings	Female recordings	Total recordings	Age range
Healthy	227	360	587	19–60
Hyperfunctional dysphonia	32	114	146	19–60
Vocal fold paresis	25	60	85	19–60

### Training and testing

To train the classifiers, we used 5-fold cross-validation. In each iteration, we held out one fold for evaluation and used the remaining folds for training. All samples from each speaker were consistently placed within a single fold to prevent the model from learning to classify voice samples based on speaker identity. We computed performance metrics based on the predictions generated for the evaluation fold. The evaluation metrics include mean accuracy and F1 score, as well as mean precision, recall, and F1 score for each class.

The dataset used in this study is imbalanced, which can be a problem for machine learning models, as they can learn to favor the majority classes and ignore the minority classes. To address this issue, we balanced the training set by oversampling the minority classes. This involved duplicating samples from the minority classes to ensure that each class had an equal number of samples in the training set, which helping to prevent the model from overfitting to the majority classes. We also applied StandardScaler to all feature embeddings to ensure that all features were on the same scale, thereby to improve the performance of the machine learning models.

## Experiments and results

This section presents a comprehensive overview of all the experiments conducted to compare the performance of the proposed voice disorders classification framework to other state-of-the-art methods. The first two experiments address binary classification problems: healthy vs. disordered and vocal fold paresis vs. hyperfunctional dysphonia. The third experiment is a multi-class classification problem.

### Healthy vs. disordered

As shown in Table 1, we have three classes: healthy, vocal fold paresis and hyperfunctional dysphonia. For this experiment, hyperfunctional dysphonia and vocal fold paresis are combined into a single class. Separate experiments have been conducted for male speakers, female speakers, and both genders combined. Table 2 shows the mean accuracy and F1 score, as well as the precision, recall, and F1 score of each class for male and female speakers. For male speakers, VGGish-SVM achieved the highest accuracy, closely followed by VGGish-EC. VGGish-SVM achieved an accuracy of 82.45%, while VGGish-EC reached 80.25%. Our method outperforms the approach presented in [45], as shown in Table 2, which uses SVM as a classifier and features extracted with wav2vec and HuBERT models, as well as SVM with MFCC and MFCC-glottal features.

**Table 2:** Performance metrics for the binary classification task of healthy vs. disordered for male and female speakers

Gender	Model	Accuracy	F1 Score	PR 0	RE 0	F1 0	PR 1	RE 1	F1 1
Male	VGGish-SVM	**82.45 ± 2.77**	82.99	0.91	0.87	0.89	0.54	0.64	0.58
	VGGish-LR	75.35 ± 4.30	75.45	0.85	0.84	0.84	0.41	0.41	0.40
	VGGish-MLP	77.09 ± 5.75	76.96	0.86	0.86	0.86	0.43	0.42	0.42
	VGGish-EC	80.25 ± 5.70	79.66	0.86	0.89	0.88	0.51	0.44	0.47
	wav2vec-SVM [45]	75.65 ± 5.81	-	0.91	0.82	0.87	0.50	0.69	0.58
	MFCC-glottal-SVM [45]	74.48 ± 5.85	-	0.90	0.84	0.87	0.51	0.64	0.57
	MFCC-SVM [45]	72.02 ± 7.75	-	0.89	0.88	0.88	0.54	0.56	0.55
	HuBERT-SVM [45]	72.14 ± 7.93	-	0.89	0.85	0.87	0.50	0.59	0.54
Female	VGGish-SVM	70.03 ± 3.07	70.05	0.79	0.77	0.77	0.53	0.57	0.54
	VGGish-LR	66.31 ± 4.86	66.68	0.77	0.72	0.74	0.48	0.55	0.51
	VGGish-MLP	68.36 ± 3.76	68.11	0.76	0.78	0.77	0.51	0.49	0.50
	VGGish-EC	71.54 ± 4.13	71.83	0.80	0.76	0.78	0.56	0.62	0.58
	wav2vec-SVM [45]	73.80 ± 5.03	-	0.84	0.77	0.80	0.60	0.71	0.65
	MFCC-glottal-SVM [45]	66.13 ± 3.11	-	0.80	0.66	0.72	0.49	0.66	0.56
	MFCC-SVM [45]	68.15 ± 4.59	-	0.81	0.68	0.74	0.51	0.68	0.58
	HuBERT-SVM [45]	**74.50 ± 4.38**	-	0.85	0.76	0.81	0.60	0.72	0.65

In the metric names, ‘0’ corresponds to the healthy class, and ‘1’ represents the disordered. PR, RE and F1 represent Precision, Recall and F1 score respectively. The mean values over folds are presented for all matrices. The highest accuracy is indicated in bold. Additionally, standard deviations for accuracy are provided

For female speakers, VGGish-EC achieved the highest accuracy with 71.54%, followed closely by VGGish-SVM, VGGish-MLP and VGGish-LR with accuracies of 70.03%, 68.36%, and 66.31% respectively. It is worth noting that this is the only case where our model demonstrates a slightly lower accuracy compared to the existing method [45], which attains its highest accuracy of 74.50% using HuBERT-SVM.

**Fig. 4:**
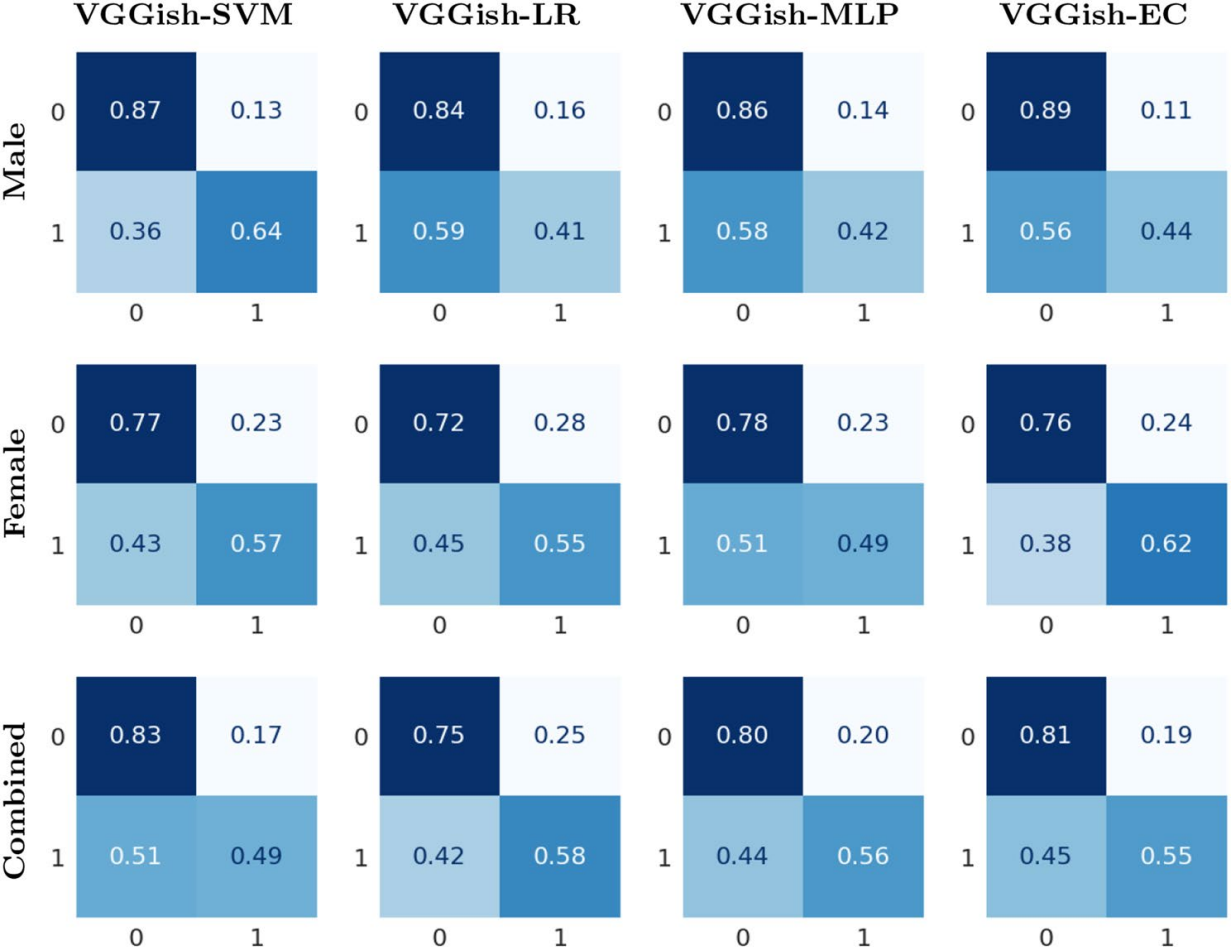
Normalized confusion matrix for healthy vs. disordered. The predicted classes are represented on the horizontal axis, while the true classes are represented on the vertical axis. Class labels: 0 for healthy and 1 for disordered

Experiments were also conducted with male and female speakers combined. The mean accuracy, F1 score, precision and recall are shown in Table 3. The results demonstrate that VGGish-EC achieved the highest overall accuracy and F1 score, with values of 73.84% and 73.92%, respectively. It was closely followed by VGGish-MLP, VGGish-SVM, and then VGGish-LR in terms of accuracy. This study’s results on combined male and female speakers can not be directly compared to those of any other study because of the differences in the datasets and disorders studied. Figure 4 presents the normalized confusion matrices for each classifier and gender.

**Table 3:** Performance metrics for the binary classification task of healthy vs. disordered for male and female speakers combined

Gender	Model	Accuracy	F1 Score	PR 0	RE 0	F1 0	PR 1	RE 1	F1 1
Male & Female	VGGish-SVM	73.35 ± 3.32	72.95	0.81	0.83	0.82	0.53	0.49	0.51
	VGGish-LR	70.05 ± 3.08	70.80	0.82	0.75	0.78	0.48	0.58	0.52
	VGGish-MLP	73.35 ± 3.93	73.57	0.82	0.80	0.81	0.53	0.56	0.54
	VGGish-EC	**73.84 ± 2.83**	73.92	0.82	0.81	0.82	0.54	0.55	0.54

In the metric names, ‘0’ corresponds to the healthy class, and ‘1’ represents the disordered. PR, RE and F1 represent Precision, Recall and F1 score respectively. The mean values over folds are presented for all matrices. The highest accuracy is indicated in bold. Additionally, standard deviations for accuracy are provided

### Hyperfunctional dysphonia vs. vocal fold paresis

To classify hyperfunctional dysphonia and vocal fold paresis, we used the same classification setup for male, female, and combined gender speakers. The mean accuracy, F1 score, precision, and recall for male and female speakers are presented in Table 4. VGGish-SVM achieved the highest accuracy (75.45%) for male speakers, while VGGish-EC achieved 71.82%. For female speakers, VGGish-EC attained the highest accuracy at 68.42%, closely followed by VGGish-SVM, VGGish-MLP, and VGGish-LR, with respective accuracies of 68.37%, 64.97%, and 62.11%. Our method outperforms the approach presented in [45]. For male speakers, their highest accuracy was 71.95%, while for female speakers, their best accuracy was 63.06%, achieved with wav2vec-SVM.

**Table 4:** Performance metrics for the binary classification task of hyperfunctional dysphonia and vocal fold paresis for male and female speakers

Gender	Model	Accuracy	F1 Score	PR 0	RE 0	F1 0	PR 1	RE 1	F1 1
Male	VGGish-SVM	**75.45 ± 6.24**	74.64	0.75	0.85	0.79	0.81	0.64	0.69
	VGGish-LR	66.52 Ł} 7.24	64.92	0.68	0.75	0.69	0.70	0.56	0.59
	VGGish-MLP	71.66 ± 11.02	70.97	0.72	0.81	0.75	0.74	0.60	0.65
	VGGish-EC	71.82 ± 7.12	70.89	0.73	0.81	0.75	0.75	0.60	0.65
	wav2vec-SVM [45]	71.95 ± 12.62	–	0.74	0.75	0.74	0.67	0.66	0.66
	MFCC-glottal-SVM [45]	69.05 ± 9.67	–	0.73	0.74	0.73	0.66	0.64	0.65
	MFCC-SVM [45]	61.60 ± 8.86	–	0.65	0.76	0.70	0.60	0.47	0.53
	HuBERT-SVM [45]	71.88 ± 10.56	–	0.73	0.80	0.76	0.70	0.62	0.66
Female	VGGish-SVM	68.37 ± 6.61	67.66	0.74	0.80	0.77	0.56	0.47	0.51
	VGGish-LR	62.11 ± 7.77	61.83	0.70	0.73	0.71	0.46	0.42	0.44
	VGGish-MLP	64.97 ± 3.51	64.96	0.74	0.72	0.73	0.50	0.52	0.50
	VGGish-EC	**68.42 ± 6.39**	68.08	0.75	0.77	0.76	0.54	0.52	0.53
	wav2vec-SVM [45]	63.06 ± 6.77	–	0.74	0.83	0.78	0.57	0.44	0.50
	MFCC-glottal-SVM [45]	59.96 ± 7.91	–	0.72	0.81	0.76	0.52	0.40	0.45
	MFCC-SVM [45]	57.09 ± 7.48	–	0.71	0.74	0.72	0.45	0.42	0.43
	HuBERT-SVM [45]	61.31 ± 5.94	–	0.73	0.78	0.75	0.52	0.45	0.48

In the metric names, ‘0’ represents hyperfunctional dysphonia class and ‘1’ represents vocal fold paresis. PR, RE and F1 represent Precision, Recall and F1 score respectively. The mean values over folds are presented for all matrices. The highest accuracy is indicated in bold. Additionally, standard deviations for accuracy are provided

**Fig. 5:**
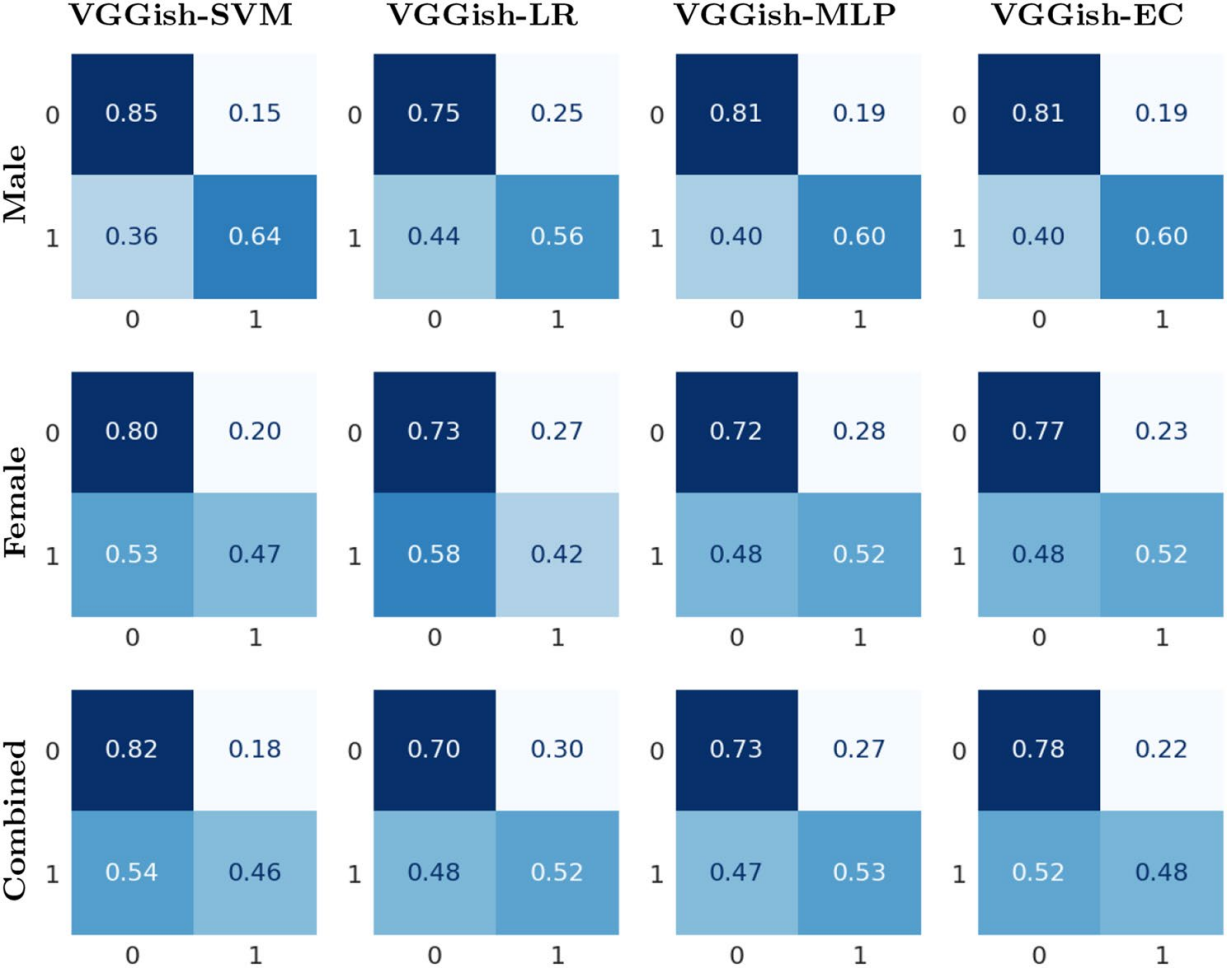
Normalized confusion matrix for hyperfunctional dysphonia vs. vocal fold paresis. The predicted classes are represented on the horizontal axis, while the true classes are represented on the vertical axis. Class labels: 0 for hyperfunctional dysphonia and 1 for vocal fold paresis

Table 5 presents the mean accuracy, F1 score, precision, and recall for male and female speakers combined. VGGish-SVM achieved the highest overall accuracy of 68.80% and F1 score of 67.64%, followed by VGGish-EC with an accuracy of 67.10% and F1 score of 66.39%. These results cannot be directly compared to previous studies because of the differences in the datasets and disorders studied. Figure 5 illustrates the normalized confusion matrices for all classifiers and genders.

**Table 5:** Performance metrics for the binary classification task of hyperfunctional dysphonia and vocal fold paresis for male and female speakers combined

Gender	Model	Accuracy	F1 Score	PR 0	RE 0	F1 0	PR 1	RE 1	F1 1
Male & Female	VGGish-SVM	**68.80 ± 6.79**	67.64	0.72	0.82	0.77	0.60	0.46	0.52
	VGGish-LR	63.20 ± 2.40	63.03	0.72	0.70	0.70	0.50	0.52	0.50
	VGGish-MLP	65.37 ± 3.08	65.27	0.73	0.73	0.72	0.53	0.53	0.53
	VGGish-EC	67.10 ± 3.93	66.39	0.72	0.78	0.75	0.57	0.48	0.52

In the metric names, ‘0’ represents hyperfunctional dysphonia class and ‘1’ represents vocal fold paresis. PR, RE and F1 represent Precision, Recall and F1 score respectively. The mean values over folds are presented for all matrices. The highest accuracy is indicated in bold. Additionally, standard deviations for accuracy are provided

### Multi-class classification

**Fig. 6:**
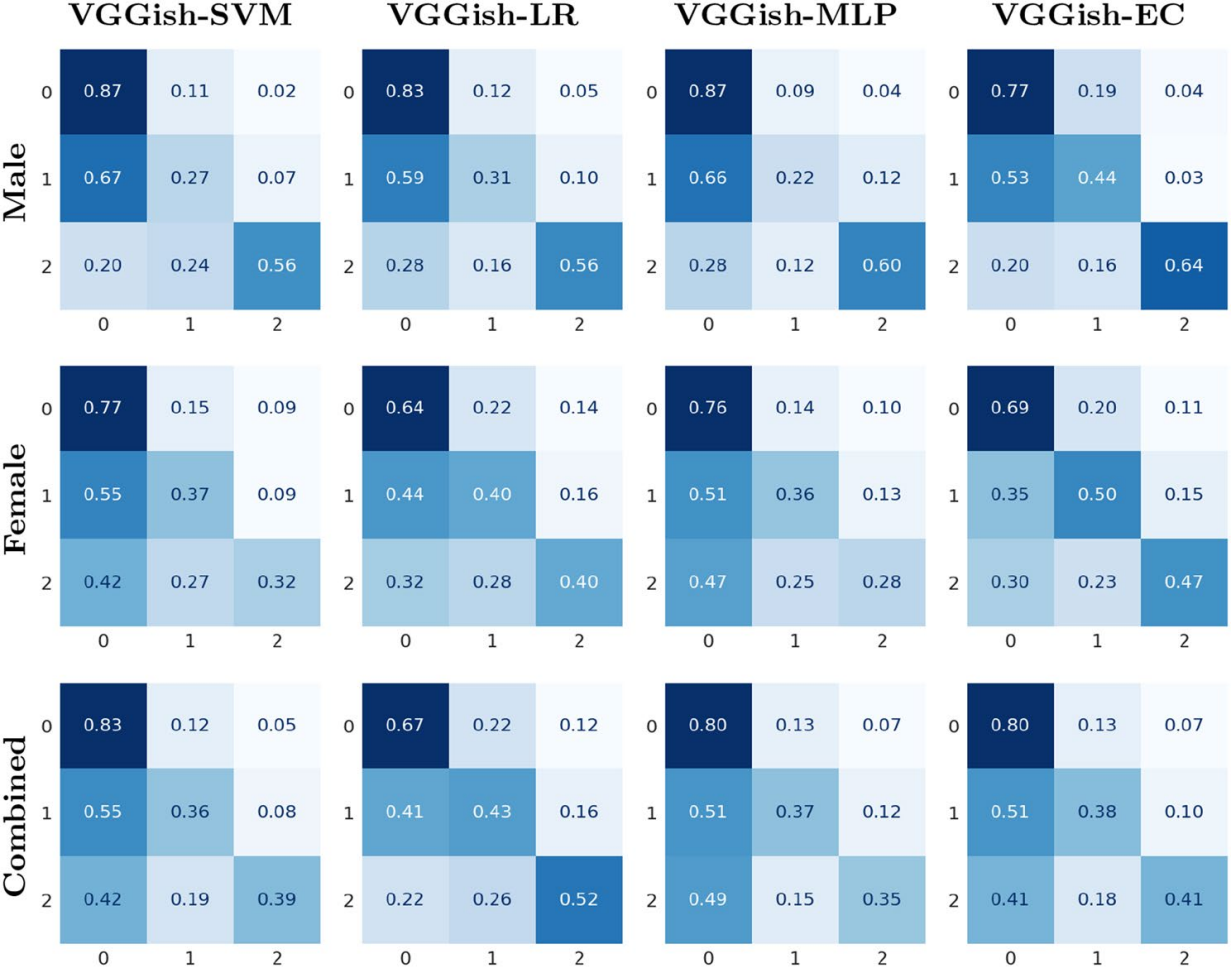
Normalized confusion matrix for multi-class classification. The predicted classes are represented on the horizontal axis, while the true classes are represented on the vertical axis. Class labels: 0 for healthy, 1 for hyperfunctional dysphonia, and 2 for vocal fold paresis

The mean accuracy, F1 score, precision, recall, and F1 score of each class for male and female speakers are shown in Table 6 and for both genders combined in Table 7. For male speakers, the highest classification accuracy achieved was 77.81%, for female speakers, it was 63.11%, and when both genders were combined, the accuracy reached 70.53%. In the case of multi-class classification, VGGish-SVM outperformed all other classifiers, including those in [41], in terms of accuracy. While the accuracy of VGGish-EC is lower than that of VGGish-SVM, it demonstrates better performance for the minority classes, which is important when dealing with imbalanced datasets, as it ensures that the model effectively recognizes and classifies the minority classes. The normalized confusion matrices for all classifiers are illustrates in Fig. 6. It is clear that the ensemble classifier enhances the performance of the minority classes for both male and female speakers.

**Table 6:** Multi-class classification performance metrics for male and female speakers

Gender	Model	Accuracy	F1 Score	PR 0	RE 0	F1 0	PR 1	RE 1	F1 1	PR 2	RE 2	F1 2
Male	VGGish-SVM	**77.81 ± 2.71**	78.34	0.88	0.87	0.88	0.20	0.27	0.22	0.73	0.56	0.62
	VGGish-LR	74.62 ± 4.86	75.83	0.88	0.83	0.85	0.23	0.31	0.25	0.58	0.56	0.55
	VGGish-MLP	77.46 ± 2.06	77.28	0.88	0.87	0.87	0.20	0.22	0.19	0.62	0.60	0.59
	VGGish-EC	72.17 ± 7.34	74.73	0.89	0.77	0.82	0.23	0.44	0.30	0.65	0.64	0.62
	wav2vec-LARGE-hier [45]	62.77 ± 10.94	-	0.92	0.79	0.85	0.30	0.55	0.39	0.47	0.53	0.50
	MFCC-glottal-SVM-hier [45]	57.35 ± 6.79	-	0.91	0.84	0.87	0.28	0.38	0.33	0.41	0.48	0.44
	MFCC-SVM-hier [45]	53.76 ± 9.35	-	0.89	0.87	0.88	0.26	0.31	0.29	0.43	0.42	0.42
Female	VGGish-SVM	**63.11** ± **3.92**	62.89	0.76	0.77	0.76	0.38	0.37	0.37	0.32	0.32	0.31
	VGGish-LR	55.99 ± 2.30	57.87	0.77	0.64	0.69	0.32	0.40	0.35	0.25	0.40	0.31
	VGGish-MLP	61.98 ± 1.94	61.64	0.76	0.76	0.76	0.38	0.36	0.37	0.24	0.28	0.26
	VGGish-EC	62.56 ± 3.41	64.28	0.82	0.69	0.75	0.39	0.50	0.44	0.33	0.47	0.38
	wav2vec-LARGE-hier [45]	55.36 ± 4.99	-	0.84	0.78	0.81	0.39	0.50	0.43	0.43	0.39	0.41
	MFCC-glottal-SVM-hier [45]	49.27 ± 5.80	-	0.80	0.65	0.72	0.30	0.49	0.37	0.37	0.33	0.35
	MFCC-SVM-hier [45]	51.11 ± 7.08	-	0.82	0.69	0.75	0.34	0.47	0.39	0.31	0.37	0.34

In the metric names, ‘0’ represents the healthy class, ‘1’ represents hyperfunctional dysphonia, and ‘2’ represents vocal fold paresis. PR, RE and F1 represent Precision, Recall and F1 score respectively. The mean values over folds are presented for all matrices. The highest accuracy is indicated in bold. Additionally, standard deviations for accuracy are provided

**Table 7:** Multi-class classification performance metrics for male and female speakers combined

Gender	Model	Accuracy	F1 Score	PR 0	RE 0	F1 0	PR 1	RE 1	F1 1	PR 2	RE 2	F1 2
Male & Female	VGGish-SVM	**70.53 ± 3.22**	69.53	0.81	0.83	0.82	0.38	0.36	0.37	0.48	0.39	0.41
	VGGish-LR	61.00 ± 6.29	63.52	0.83	0.67	0.74	0.30	0.43	0.35	0.34	0.52	0.40
	VGGish-MLP	67.85 ± 2.65	67.80	0.80	0.80	0.80	0.38	0.37	0.37	0.36	0.35	0.35
	VGGish-EC	68.34 ± 3.45	68.52	0.81	0.80	0.80	0.38	0.38	0.38	0.40	0.41	0.40

In the metric names, ‘0’ represents the healthy class, ‘1’ represents hyperfunctional dysphonia, and ‘2’ represents vocal fold paresis. PR, RE and F1 represent Precision, Recall and F1 score respectively. The mean values over folds are presented for all matrices. The highest accuracy is indicated in bold. Additionally, standard deviations for accuracy are provided

## Discussion

The proposed voice disorders classification system demonstrates superior performance compared to state-of-the-art methods. In this study, we employed machine learning classifiers, particularly ensemble classifiers, to evaluate high-level feature embeddings extracted using a pre-trained VGGish model. To evaluate the effectiveness of our approach, the results were compared with those reported in [45], where the same dataset was used for evaluation. Our study shows that extracting features with a pre-trained model outperforms MFCC feature-based systems, which are the most commonly used features in the detection and classification of voice disorders [46–48]. This statement is also confirmed by [45], where the authors extract features with the wav2vec and HuBERT models and compare the results with MFCC features.

Our study also investigated the performance of the proposed system on male and female speakers separately for both binary and multi-class classification tasks. Interestingly, our findings reveal a consistent trend where the accuracy of male speakers outperforms that of female speakers. The best accuracy for healthy vs. disordered classification was 82.45% for male speakers and 71.54% for female speakers. Similarly, the highest accuracy for hyperfunctional dysphonia vs. vocal fold paresis classification was 75.45% for male speakers and 68.42% for female speakers. In the multi-class classification scenario, the accuracy differences between male and female speakers continued similar trends. For male speakers, our model achieved an impressive accuracy of 77.81%, however, for female speakers, the highest accuracy observed was 63.11%. It is important to highlight that the binary classification of healthy vs. disordered voices for female speakers stands as the only case where our model exhibited a slightly lower accuracy compared to the results reported in [45].

VGGish-SVM achieved the highest accuracy for male speakers and VGGish-EC for female speakers in both binary classification tasks (i.e., healthy vs. disordered and hyperfunctional dysphonia vs. vocal fold paresis). In multi-class classification, VGGish-SVM performed better for both genders. However, while VGGish-EC achieved a lower overall accuracy than VGGish-SVM in multi-class classification, it outperformed VGGish-SVM on minority classes. For example, for male speakers, the precision and recall for hyperfunctional dysphonia with VGGish-SVM were 0.20% and 0.27%, respectively, while with VGGish-EC, the precision and recall were 0.23 and 0.44, respectively. Similarly, VGGish-EC performed better for vocal fold paresis. The same trend was observed for female speakers. In multi-class classification, for male speakers, the lowest F1 score is recorded for hyperfunctional dysphonia, while for female speakers, the lowest F1 score is observed for vocal fold paresis. These classes presented particular challenges in terms of accuracy, probably because of the smaller number of samples available for these classes. This underlines the importance of addressing data imbalance in future research to further enhance classification performance.

As part of our future work, we plan to incorporate explainability techniques such as LIME, SHAP, and Grad-CAM. These methods will enable us to better understand the contribution of different features in the classification process and provide visual insights into the regions of the spectrograms that are most influential in decision-making. It will help build trust in the model’s predictions and facilitate its integration into diagnostic workflows.

This study demonstrates the efficacy of hybrid frameworks for voice disorder classification using controlled datasets. However, it does not evaluate real-time performance, which is a critical factor for clinical deployment. Furthermore, the computational demands of the VGGish feature extractor and classifier pipeline may introduce latency in unoptimized implementations. Future work will focus on optimizing the framework for low-latency inference (e.g., via model lightweighting, edge-device deployment) and validating its performance on streaming audio data acquired in clinical or telehealth settings.

## Conclusion

In this paper, we proposed a two-stage hybrid framework for voice disorders classification. In the first stage, we utilized a pre-trained VGGish model to extract high-level feature embeddings from the log-mel spectrograms of voice data. In the second stage, we evaluated four classifiers: support vector machine (SVM), logistic regression (LR), multilayer perceptron (MLP), and ensemble classifier.

The results of our study demonstrate the potential of using a pre-trained VGGish model to extract features for voice disorders classification. We achieved state-of-the-art results on the SVD dataset, outperforming the baseline systems that used MFCC features, MFCC-glottal features, as well as features extracted with pre-trained wav2vec and HuBERT models. Compared to the best baseline accuracy, we improved by 6.8% for male speakers in healthy vs. disordered task, 3.5% and 5.36% for male and female speakers respectively in hyperfunctional dysphonia vs. vocal fold paresis tasks. In the context of multi-class classification, our method significantly outperformed the baseline, achieving a 15.04% improvement for male speakers and a 7.75% improvement for female speakers.

While our model excelled in most scenarios, there was a slight exception. In the healthy vs. disordered task for female speakers, our model demonstrated an accuracy that was 2.96% lower when compared to the baseline. The accuracies for the combined dataset of male and female speakers are also promising in all three scenarios. It is important to note that these combined results cannot be directly compared to existing studies because of variations in the datasets and the types of voice disorders investigated.

In binary classification, VGGish-SVM exhibited the highest accuracy for male speakers, while VGGish-EC performed best for female speakers. However, in multi-class classification, VGGish-SVM outperformed other models for both genders. Notably, VGGish-EC demonstrated its strength in handling minority classes, an important aspect of medical applications. The results confirm that VGGish-EC provides more balanced accuracy by giving importance to the minority classes. Although we used oversampling to balance the classes, the accuracy of minority classes remains comparatively lower. Future research will focus on improving the robustness and generalizability of the proposed two-stage hybrid framework for voice disorders classification. Additionally, expanding the dataset to include a more diverse and a broader range of voice disorders will be crucial for enhancing the model’s applicability in real-world scenarios.

## Data Availability

The data used in this study were selected from the publicly available Saarbruecken Voice Database (SVD). The full database can be accessed at the link: https://stimmdatenbank.coli.uni-saarland.de/.
